# Computer Vision in Healthcare Applications

**DOI:** 10.1155/2018/5157020

**Published:** 2018-03-04

**Authors:** Junfeng Gao, Yong Yang, Pan Lin, Dong Sun Park

**Affiliations:** ^1^College of Biomedical Engineering, South-Central University for Nationalities, Wuhan 430074, China; ^2^Key Laboratory of Cognitive Science, State Ethnic Affairs Commission, Wuhan 430074, China; ^3^Hubei Key Laboratory of Medical Information Analysis and Tumor Diagnosis & Treatment, Wuhan 430074, China; ^4^School of Information Technology, Jiangxi University of Finance and Economics, Nanchang 330032, China; ^5^IT Convergence Research Center, Chonbuk National University, Jeonju, Jeonbuk 54896, Republic of Korea

The research of computer vision, imaging processing and pattern recognition has made substantial progress during the past several decades. Also, medical imaging has attracted increasing attention in recent years due to its vital component in healthcare applications. Investigators have published a wealth of basic science and data documenting the progress and healthcare application on medical imaging. Since the development of these research fields has set the clinicians to advance from the bench to the bedside, the *Journal of Healthcare Engineering* set out to publish this special issue devoted to the topic of advanced computer vision methods for healthcare engineering, as well as review articles that will stimulate the continuing efforts to understand the problems usually encountered in this field. The result is a collection of fifteen outstanding articles submitted by investigators.

Following the goal of special issue, we identify four major domains covered by the papers. The first is medical image analysis for healthcare, the second is the computer vision for predictive analytics and therapy, the third is fundamental algorithms for medical images, and the last one focuses on the machine learning algorithms for medical images. Here, we give the review of these published papers.

## 1. Analysis of Medical Image

This theme attempts to address the improvement and new techniques on the analysis methods of medical image. First, integration of multimodal information carried out from different diagnostic imaging techniques is essential for a comprehensive characterization of the region under examination. Therefore, image coregistration has become crucial both for qualitative visual assessment and for quantitative multiparametric analysis in research applications. S. Monti et al. in Italy “An Evaluation of the Benefits of Simultaneous Acquisition on PET/MR Coregistration in Head/Neck Imaging” compare and assess the performance between the traditional coregistration methods applied to PET and MR acquired as single modalities and the obtained results with the implicitly coregistration of a hybrid PET/MR, in complex anatomical regions such as the head/neck (HN). The experimental results show that hybrid PET/MR provides a higher registration accuracy than the retrospectively coregistered images.

The feature extraction is one of the key issues for the analysis of medical images. I. I. Esener et al. in Turkey “A New Feature Ensemble with a Multistage Classification Scheme for Breast Cancer Diagnosis” develop a new and effective feature ensemble with a multistage classification which is used in a computer-aided diagnosis (CAD) system for breast cancer diagnosis. In this new method, four features, the local configuration pattern-based, statistical, and frequency domain features were concatenated as feature vectors, and eight well-known classifiers are used in a multistage classification scheme. High classification accuracy was obtained, and it shows that the proposed multistage classification scheme is more effective than the single-stage classification for breast cancer diagnosis.

Currently, the traditional approach to reduce colorectal cancer-related mortality is to perform regular screening in search for polyps, which results in polyp miss rate and inability to perform visual assessment of polyp malignancy. D. Vazquez et al. in Spain and Canada “A Benchmark for Endoluminal Scene Segmentation of Colonoscopy Images” propose an extended benchmark of colonoscopy image segmentation and establish a new strong benchmark for colonoscopy image analysis. By training a standard fully convolutional networks (FCN), they show that in endoluminal scene segmentation, the performance of FCN is better than the result of the prior researches.

## 2. Computer Vision for Predictive Analytics and Therapy

Computer vision technique has shown great application in surgery and therapy of some diseases. Recently, three-dimensional (3D) modeling and rapid prototyping technologies have driven the development of medical imaging modalities, such as CT and MRI. P. Gargiulo et al. in Iceland “New Directions in 3D Medical Modeling: 3D-Printing Anatomy and Functions in Neurosurgical Planning” combine CT and MRI images with DTI tractography and use image segmentation protocols to 3D model the skull base, tumor, and five eloquent fiber tracts. The authors provide a great potential therapy approach for advanced neurosurgical preparation.

The elderly is easy to fall and it will harm the body and accordingly has serious negative mental impacts on them. T.-H. Lin et al. in Taiwan “Fall Prevention Shoes Using Camera-Based Line-Laser Obstacle Detection System” design an interesting line-laser obstacle detection system to prevent the elderly from falls. In the system, a laser line passes through a horizontal plane and has a specific height to the ground, and optical axis in a camera has a specific inclined angle to the plane, and hence, the camera can observe the laser pattern to obtain the potential obstacles. Unfortunately, this system designed is useful mainly for indoor applications instead of outdoor environment.

Human activity recognition (HAR) is one of the widely studied computer vision problem. S. Zhang et al. in China “A Review on Human Activity Recognition Using Vision-Based Method” introduce an overview of various HAR approaches as well as their evolutions with the representative classical literatures. The authors highlight the advances of image representation approaches and classification methods in vision-based activity recognition. Representation approaches generally include global representations, local representations, and depth-based representations. They accordingly divide and describe the human activities into three levels including action primitives, actions/activities, and interactions. Also, they summarize the classification techniques in HAR application which include 7 types of method from the classic DTW and the newest deep learning. Lastly, they address that applying these current HAR approaches in real-world systems or applications has great challenge although up to now recent HAR approaches have achieved great success. Also, three future directions are recommended in their work.

## 3. Fundamental Algorithms for Medical Images

The majority of this issue focuses on the research of improved algorithm for medical images. Organ segmentation is a prerequisite for CAD systems. In fact, the segmentation algorithm is the most important and basic for image processing and also enhance the level of disease prediction and therapy. C. Pan et al. in China “Leukocyte Image Segmentation Using Novel Saliency Detection Based on Positive Feedback of Visual Perception” use the ensemble of polyharmonic extreme learning machine (EPELM) and positive feedback of perception to detect salient objects, which is totally data-driven without any prior knowledge and labeled samples compared with the existed algorithms. A positive feedback module based on EPELM focuses on fixation area for the purpose of intensifying objects, inhibiting noises, and promoting saturation in perception. Experiments on several standard image databases show that the novel algorithm outperforms the conventional saliency detection algorithms and also segments nucleated cells successfully in different imaging conditions.

High-intensity focused ultrasound (HIFU) has been proposed for the safe ablation of both malignant and benign tissues and as an agent for drug delivery, while MRI has been proposed for guidance and monitoring for the therapy. A. Vargas-Olivares et al. in México and Canada “Segmentation Method for Magnetic Resonance-guided High-Intensity Focused Ultrasound Therapy Planning” used the MR images for the HIFU therapy planning and propose an efficient segmentation approach. The segmentation scheme uses the watershed method to identify the regions found on the HIFU treatment. In addition, the authors also propose a thread pool strategy, in order to reduce the computational overload of the processing time of the group of MR images and the segmentation algorithm.

Recently, random walkers (RW) have attracted a growing interest to process segmentation of medical images. However, classical RW method needs a long computation time and a high memory usage because of the construction of corresponding large-scale graph to solve the resulting sparse linear system. C. Dong et al. in China and USA “An Improved Random Walker with Bayes Model For Volumetric Medical Image Segmentation” incorporate the prior (shape and intensity) knowledge in the optimization of sparse linear system. Integrating the Bayes model into the RW sparse system, the organ is automatically segmented for the adjacent slice, which is called RWBayes algorithm in the article. Compared with the conventional RW and the state-of-the-art interactive segmentation methods, their method can significantly improve the segmentation accuracy and could be extended to segment other organs in the future.

Automatic segmentation of the spinal cord in MR images remains a difficult task. C.-C. Liao et al. in Taiwan “Atlas-Free Cervical Spinal Cord Segmentation on Midsagittal T2-Weighted Magnetic Resonance Images” present an automatic segmentation method on sagittal T2-weighted images. The method is atlas-free, in which expectation maximization algorithm is used to cluster the pixels on a midsagittal MR image according to their gray levels or SIs. Dynamic programming is used to detect anatomical structures and their edges. The detection of the anterior and posterior edges of the spinal cord within the cervical spinal canal is finally successful in all 79 images, showing its high accuracy and robustness. Based on this proposed algorithm, using alone or combining with others, one can develop a computer-aided diagnosis system with massive screening on cervical spine diseases. Finally, the authors point out several limitations in the algorithm, such as its inability to be applied to lower lumbar spinal levels.

The misalignments originated from motion and deformation often result in errors in estimating an apparent diffusion coefficient (ADC) map fitted with prostate DWI, and the ADC map is an important indicator in diagnosing prostate cancer. Until now, there are few studies that focus on this misalignment in prostate DWI. L. Hao et al. in China “Nonrigid Registration of Prostate Diffusion-Weighted MR” apply affine transformation to DWI to correct intraslice motions. Then, nonrigid registration based on free-form deformation (FFD) is used to compensate for intraimage deformations. The experimental results show that the proposed algorithm can correct the misalignment of prostate DWI and decrease the artifacts of ROI in the ADC maps. These ADC maps thus obtain sharper contours of lesions, which are helpful for improving the diagnosis and clinical staging of prostate cancer.

Medical ultrasound is widely used in the diagnosis and assessment of internal body structures and also plays a key role in treating various diseases due to its safety, noninvasion, and well tolerance in patients. However, the images are always contaminated with speckle noise and hence hinder the identification of image details. Currently, many methods have been proposed to remove the noise and preserve the image details at the same time. M. Szczepański and K. Radlak in Poland “Digital Path Approach Despeckle Filter for Ultrasound Imaging and Video” propose a so-called escaping paths based on traditional digital paths, and also, they extend this concept from the spatial domain (2D) to the spatiotemporal domain (3D) that is designed for multiplicative noise suppression, specifically for ultrasound image and video filtering. In addition, the extended neighborhood model is used to increase the filter denoising ability, which is based on von Neumann concept derived from cellular automata theory. The experimental results prove that the proposed removal technique outperforms the state-of-the-art approach for multiplicative noise removal with lower computational overload which enables one to complete image processing tasks and image enhancement of video streams in a real-time environment.

A primary challenge in accelerating MR imaging is how to reconstruct high-resolution images from undersampled *k*-space data. There is a trade-off between the spatial resolution and temporal resolution. J. Chen et al. in China “Low-Rank and Sparse Decomposition Model for Accelerating Dynamic MRI Reconstruction” introduce a low-rank and sparse decomposition model to resolve this problem, which is based on the theory of robust principal component analysis (RPCA). Unlike *k-t* RPCA (a method that uses the low-rank plus sparse decomposition prior to reconstruction of dynamic MRI from part of the *k*-space measurements), the authors propose inexact augmented Lagrangian method (IALM) to solve the optimization of RPCA and to accelerate the dynamic MRI reconstruction from highly undersampled *k*-space data, which has a generalized formulation capability of separating dynamic MR data into low-rank and sparse component. The experimental results on cardiac datasets prove that the proposed method can achieve more satisfactory reconstruction performance and faster reconstruction speed, compared with the state-of the-art reconstruction methods.

## 4. Machine Learning Algorithms for Medical Images

The growth of the older adult population in the world is surprising and it will have a great impact on the healthcare system. The elders always lack self-care ability and hence, healthcare and nursing robot draw much attention in recent years. Although somatosensory technology has been introduced into the activity recognition and healthcare interaction of the elderly, traditional detection method is always in a single modal. In order to develop an efficient and convenient interaction assistant system for nurses and patients with dementia, X. Dang et al. in China “An Interactive Care System Based on a Depth Image and EEG for Aged Patients with Dementia” propose two novel multimodal sparse autoencoder frameworks based on motion and mental features. First, the motion is extracted after the preprocessing of depth image and then EEG signals as the mental feature is recorded. The proposed novel system is designed to be based on the multimodal deep neural networks for the patient with dementia with special needs. The input features of the networks include (1) extracted motion features based on the depth image sensor and (2) EEG features. The output layer is the type recognition of the patient's help requirement. Experimental results show that the proposed algorithm simplifies the process of the recognition and achieved 96.5% and 96.4% (accuracy and recall rate), respectively, for the shuffled dataset, and 90.9% and 92.6%, respectively, for the continuous dataset. Also, the proposed algorithms simplify the acquisition and data processing under high action recognition ratio compared with the traditional method.

Recently, deep learning has become very popular in artificial intelligence. Q. Song et al. in China “Using Deep Learning for Classification of Lung Nodules on Computed Tomography Images” employ a convolution neural network (CNN), deep neural network (DNN), and stacked autoencoder (SAE) for the early diagnosis of lung cancer to doctors. The experimental results suggest that the CNN archived the best performance than DNN and SAE.

N. D. Kamarudin et al. in Malaysia and Japan “A Fast SVM-Based Tongue's Colour Classification Aided by *k*-Means Clustering Identifiers and Colour Attributes as Computer-Assisted Tool for Tongue Diagnosis” propose a two-stage classification system for tongue color diagnosis aided with the devised clustering identifiers, and it can diagnose three tongue colors: red, light red, and deep red. The diagnosis system is very useful for the early detection of imbalance condition inside the body. Experimental result shows that this novel classification system outperforms the conventional SVM by 20% computational time and 15% in terms of classification accuracy.

## 5. Conclusion

These authors highlight both the promise and the challenges faced by this healthcare application field of medical images. Their researches identify the critical need for clinical and theory prospective of medical images. This special issue brings about various new developments in computer vision about medical images and clinical application. In summary, this special issue provides a snapshot of the computer vision in healthcare applications on medical images across the globe. Hopefully, this publication will provide a good reference for future computer vision, analysis algorithms, and machine learning of medical images. However, there are still some key messages that emerge from the papers compiled within this special issue: there still remain limitation and challenge for computer vision and various algorithms and processing techniques of medical images although these works show good efficiency than traditional and state-of-art methods. We hope that this theme issue will further advance our understanding of computer vision about medical image processing and healthcare applications and pave the way for new directions in medical images and computer vision research across health and disease.

